# Biphasic regulation of A20 gene expression during human cytomegalovirus infection

**DOI:** 10.1186/1743-422X-11-124

**Published:** 2014-07-08

**Authors:** Su Yeon Gu, Young-Eui Kim, Ki Mun Kwon, Tae-Hee Han, Jin-Hyun Ahn

**Affiliations:** 1 Department of Molecular Cell Biology, Samsung Biomedical Research Institute, Sungkyunkwan University School of Medicine, 2066 Seoburo, Suwon 440-746, Republic of Korea

**Keywords:** HCMV, A20, NF-κB, IE1

## Abstract

**Background:**

The A20 ubiquitin-editing enzyme is a target of nuclear factor kappa B (NF-κB) and also plays a key role in regulating the NF-κB signaling pathway. NF-κB activity is increased during human cytomegalovirus (HCMV) infection and HCMV appears to be adapted to this change. To better understand the regulation of NF-κB signaling during HCMV infection, we investigated how A20 expression is controlled during HCMV infection.

**Methods:**

The expression level of A20 in human fibroblast cells infected with HCMV or UV-inactivated virus (UV-HCMV) was measured by immunoblot analysis, cell staining, and quantitative real-time PCR. Changes of histone modifications on the A20 promoter were determined by chromatin immunoprecipitation assays. Lentiviral vectors were used to knockdown A20 in fibroblast cells.

**Results:**

A20 expression was increased at early times after HCMV infection. This increase of the A20 protein level was promoted by viral gene expression under low viral load conditions. The viral IE1 protein, which is known to activate NF-κB, increased the A20 promoter activity through the upstream NF-κB sites in reporter assays, suggesting that IE1 is at least partly involved in A20 induction. Analysis of A20 expression with a high viral load demonstrated that the A20 regulation by HCMV was biphasic; both A20 protein and mRNA levels were increased at the early stage of infection, but decreased at the late stage. Under high viral load conditions, A20 upregulation was more profound with UV-HCMV than with HCMV, indicating a role of the viral gene product(s) in limiting A20 induction. Consistently, more histone modifications for euchromatin were found on the A20 promoter during UV-HCMV infection than with HCMV infection. A20 knockdown by shRNA reduced HCMV growth.

**Conclusion:**

These results suggest that the biphasic regulation of A20 expression may be important for productive HCMV infection.

## Background

The nuclear factor-kappa B (NF-κB) signaling pathway controls inflammation, the immune response, and cell survival during viral infections. The regulation of NF-κB signaling during human cytomegalovirus (HCMV) infection is complicated. The pro-viral role of NF-κB for HCMV gene expression is suggested by the presence of the NF-κB sites on the viral major immediate-early (IE) promoter, and the IE1 protein autoregulates its own promoter through upstream NF-κB sequences [1]. HCMV appears to activate NF-κB at different levels. For example, NF-κB is highly induced in HCMV-infected fibroblasts [2]. Binding of viral glycoproteins to cell receptors activates NF-κB in fibroblasts and monocytes [3,4]. In addition, NF-κB upregulation is mediated by viral IE proteins through induction of the cellular factor, Sp1, which activates the NF-κB p105/p50 and p65 subunit promoters [3,5]. The role of IE1 in the induction of NF-κB is demonstrated in vascular smooth muscle cells, where IE1 selectively induces nuclear RelB and p50 among NF-κB/Rel factor [6], particularly through Jun kinase and c-Jun/Fra-2 AP-1 complexes for RelB induction [7].

Since NF-κB activity leads to an increase in antiviral inflammatory cytokine expression, an antiviral role for NF-κB signaling during viral infection is expected. When NF-κB activation is blocked, cytokines that inhibit HCMV are no longer produced in culture supernatants, and overexpression of IκB kinase β (IKKβ) inhibits HCMV replication [8]. Thus, viruses appear to establish several strategies to interfere with innate immunity by preventing NF-κB pathway activation [9]. IE2 inhibits tumor necrosis factor α-induced NF-κB DNA binding, leading to suppression of the IFN-β promoter [10].

A20, a ubiquitin-editing enzyme that belongs to the OTU domain family of deubiquitinases (DUBs), is induced by NF-κB and also acts as a negative regulator of NF-κB signaling, playing a critical role in terminating NF-κB responses to various stimuli [11]. A20 DUB activity cleaves K63-linked poly-ubiquitin chains from RIP1 [12] and TRAF6 [13]. In addition, A20 harbors atypical zinc finger-dependent K48-specific E3 ubiquitin ligase activity that adds K48-linked poly-ubiquitin chains to RIP1, inducing its degradation [12]. A20 also promotes proteasomal degradation of ubiquitin E2 conjugating enzymes, Ubc13 and UbcH5, involved in NF-κB signaling [14]. By targeting IκB kinase γ (IKKγ), A20 also non-catalytically inhibits TNFR-mediated signaling [15]. The importance of A20 in NF-κB regulation is shown in *in vivo* studies demonstrating that A20-deficiency in mice results in excess NF-κB activity and increased inflammation in several organs [13,16]. Furthermore, A20 is reported to stabilize NF-κB-inducing kinase (NIK), promoting the transition from canonical to non-canonical NF-κB activation for proper immune system control [17].

It has been shown that A20 is induced in human monocytes after HCMV infection and that the binding of viral glycoproteins to cellular receptors plays a role in this induction [4]. In the present study, we investigated how A20 expression is regulated in human fibroblasts after HCMV infection. We showed that A20 expression is biphasically regulated during HCMV infection. A20 expression was initially increased at early times of infection but inhibited at later stages. We also found that the viral IE1 protein activates the A20 promoter through the upstream NF-κB biding sites, suggesting a role of IE1 in the initial A20 activation. Analysis of A20 expression with a high viral load demonstrated that the newly synthesized viral gene product(s) epigenetically limit the upregulation of A20 transcription. We also found that A20 knockdown reduces HCMV growth, suggesting that the biphasic regulation of A20 expression may be important for productive HCMV infection.

## Results

### Effect of HCMV infection on A20 expression

**Figure 1:**
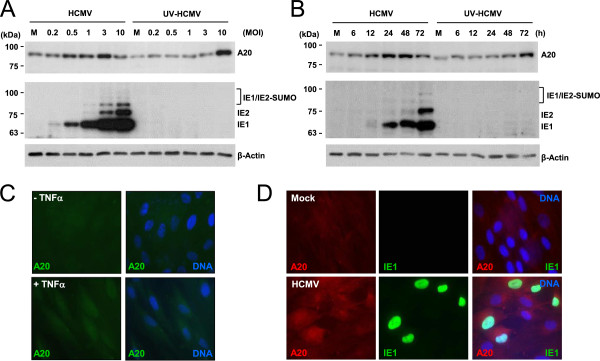
**Effect of HCMV infection on A20 protein level. (A)** HF cells were infected with HCMV or UV-inactivated virus (UV-HCMV) at the indicated MOIs. Total cell lysates were prepared at 24 h and immunoblotted with anti-A20 and anti-β-actin Abs. **(B)** HF cells were infected with HCMV or UV-HCMV at an MOI of 0.5 for the indicated times, followed by immunoblotting as in (A). **(C)** HF cells were untreated or treated with TNF-α (50 ng/ml) for 2 h. Cells were fixed with the paraformaldehyde procedure and IFA was performed with anti-A20 Ab. A mounting solution containing Hoechst dye was used to stain cell nuclei. FITC-conjugated anti-mouse IgG and rhodamine/Red X-coupled anti-rabbit IgG Abs were used for visualization. **(D)** HF cells were mock-infected or infected with HCMV at an MOI of 0.5 for 24 h. Cells were fixed and stained with anti-A20 and anti-IE1 Abs as in (C). For visualization, rhodamine/Red X-coupled anti-mouse IgG and FITC-conjugated anti-rabbit IgG Abs were used.

We investigated how the A20 protein level is regulated during HCMV infection. HF cells were infected with HCMV or UV-inactivated virus (UV-HCMV) at different multiplicities of infection (MOI) (from 0.2 to 10) and the A20 protein level at 24 h after infection was measured by immunoblotting. The A20 level was increased by HCMV infection at an MOI of 0.2, 0.5, 1, and 3, showing peak induction at an MOI of 3, but this increase was reduced at an MOI of 10. UV-HCMV infection less effectively upregulated the A20 level than HCMV at relatively low MOIs (from 0.2 to 3); however, it more effectively upregulated A20 than HCMV at an MOI of 10 (Figure 1A). We also examined the time-course effect of HCMV infection on the A20 level at an MOI of 0.5. The results showed that the A20 level was increased from the early phases of infection. UV-HCMV less effectively upregulated the A20 level than HCMV during the early phases, whereas it more effectively upregulated the A20 level than HCMV at 72 h (Figure 1B). Considering that the MOI-dependent difference often reflects the progression of infection, these results suggest that A20 expression is induced at the early phases of infection, but this induction may be downregulated at the late phases. Our analysis with UV-HCMV also suggests that the newly expressed viral gene product(s) may promote A20 expression at early times under low MOI conditions.The A20 levels determined by immunobot analysis reflect the mean values of A20 proteins of the whole cell population. To examine the induction of A20 by HCMV infection at the single cell level, we infected HF cells with HCMV at an MOI of 0.5 for 24 h and stained the cells for A20 and IE1. The results of IFA showed that the A20 signals were increased in virus-infected (IE1-positive) cells in both the nucleus and cytoplasm to a higher level than in cells treated with TNFα (Figure 1C and D). Together, these results support the idea that HCMV infection results in a modest increase in A20 protein level during the early stage of virus infection.

### Evaluation of the IE1-mediated NF-κB activation on A20 promoter activity

**Figure 2:**
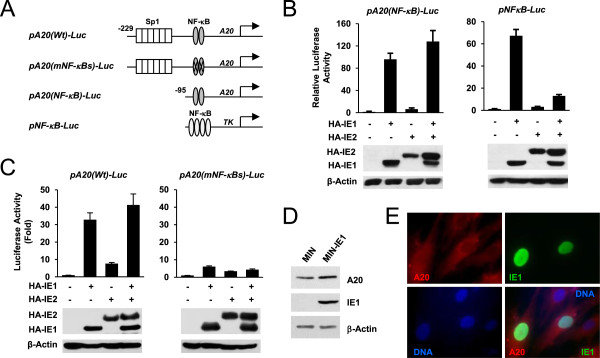
**IE1-mediated activation of the A20 promoter in reporter assays. (A)** Schematic representation of the NF-κB reporter genes used in reporter assays (see Materials and Methods). The binding sites for Sp1 and NF-κB are indicated. TK indicates the minimal core region of the HSV-1 TK promoter. **(B)** Via electroporation, HF cells were cotransfected with 0.5 μg of reporter plasmids containing the A20(NF-κB)-luciferase or the synthetic NF-κB-luciferase reporter gene (pNFκB-Luc) and 0.5 μg of plasmids expressing HA-IE1 or HA-IE2, as indicated. At 48 h, total cell lysates were prepared, and luciferase assays were performed. The levels of IE1, IE2, and β-actin are shown by immunoblotting with anti-HA and anti-β-actin Abs. **(C)** HF cells were cotransfected with an A20-luciferase reporter plasmid containing wild-type or mutant NF-κB sites [pA20(Wt)-Luc or pA20(mNF-κBs)-Luc] and plasmids expressing HA-IE1 or HA-IE2, and the luciferase assays and immunoblot analysis were performed. **(D and E)** Control HF cells (MIN) or cells expressing IE1 (MIN-IE1) were produced by retroviral transduction. Total cell lysates were prepared and immunoblot analysis were performed with mouse MAbs for A20, IE1 (810R) or β-actin **(D)**. Cells were fixed in methanol and double-alabel IFA was performed with anti-A20 and anti-IE1 Abs. Rhodamine/Red X-coupled anti-mouse IgG and FITC-conjugated anti-rabbit IgG were used for visualization **(E)**.

Since HCMV IE1 induces NF-κB activity, it is expected that the A20 promoter, which contains the NF-κB binding sequences, is also activated by IE1. To test this, transient transfection reporter gene assays were performed in HCMV-permissive HF cells using the luciferase reporter plasmid pA20(NF-κB)-Luc, which contains the A20 promoter region (from -95 to +9) with two NF-κB sites, and a synthetic pNFκB-Luc plasmid, which contains four copies of NF-κB sites (Figure 2A). We found that IE1 substantially increased the activity of both the A20 promoter and the synthetic promoter (Figure 2B). HCMV IE2 did not significantly affect either promoter, and when both IE1 and IE2 were expressed together, IE2 did not affect IE1-mediated A20 promoter activation, although it interfered with the IE1 activity for the NF-κB synthetic promoter (Figure 2B). We also tested a distinct set of A20 reporter plasmids; pA20(Wt)-Luc, which contains six Sp1 sites and NF-κB sites, and its NF-κB site mutant version, pA20 (mNF-κBs)-Luc (Figure 2A). The results of similar transfection assays showed that IE1 activated the wild-type A20 promoter by 32-fold but upregulated the NF-κB mutant version by only 6-fold, whereas IE2 did not significantly affect IE1 activity (Figure 2C). These results indicate that IE1 activates the A20 promoter through the NF-κB sites in reporter assays.The effect of IE1 expression on A20 expression was further investigated using IE1-expressing HF cells, which were produced by retroviral vector transduction. We found that the A20 level was higher in IE1-expressing cells than in control cells in both immunoblot assays (Figure 2D) and IFA (Figure 2E). The A20 unpregulated by IE1 transfection appeared to accumulate largely in the cytoplasm. A similar localization pattern of A20 was observed in IE1-expressing cells after TNFα treatment (data not shown). Together with the results of reporter assays, these results indicate that IE1 is at least partly involved in A20 induction.

### Biphasic regulation of A20 expression by HCMV at a high viral load

**Figure 3:**
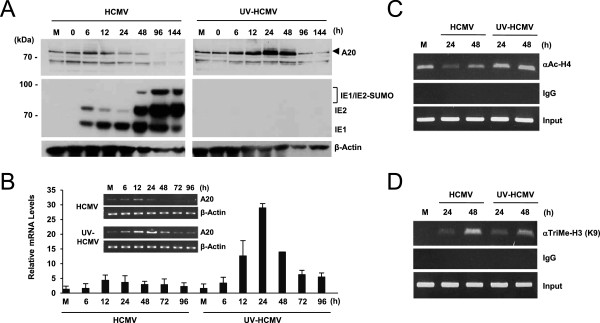
**Comparison of A20 protein and mRNA levels and histone modifications on the A20 promoters between HCMV and UV-HCMV infection at a high MOI. (A)** HF cells were infected with HCMV or UV-HCMV at an MOI of 5 for the indicated times, and the A20 protein levels were analyzed by immunoblotting. The progression of the infection with HCMV and the lack of IE1 and IE2 expression with UV-HCMV were shown by detecting the levels of IE1 and IE2. **(B)** HF cells were infected as in (A) and total RNAs were prepared at the indicated time points. The A20 mRNA levels were determined by qRT-PCR. The qRT-PCR results are shown as the mean values and standard errors of three independent experiments. The insert shows the RT-PCR products of A20 and β-actin (a loading control). **(C and D)** HF cells were infected with HCMV or UV-HCMV at an MOI of 5. Samples were prepared at the indicated time points, and ChIP assays were performed using Abs specific for acetylated histone H4 (αAc-H4) **(C)** and tri-methylated histone H3 (Lys9) [αTriMe-H3 (K9)] **(D)**. The amounts of coprecipitated DNA were determined by PCR. ChIP with non-specific immunoglobulin G (IgG) was used as a negative control.

We next investigated the effect of HCMV infection on A20 expression under high viral load conditions. When HF cells were infected with HCMV at an MOI of 5, the A20 protein level was initially increased until 6 to 12 h after infection, but it was subsequently decreased to below the basal level of mock-infected cells (Figure 3A). UV-HCMV also showed a similar pattern of initial increase and subsequent decrease in A20 protein levels, but the increase was more profound and sustained than in HCMV, leading to maximal expression at 24 or 48 h after infection (Figure 3A). The progression of HCMV infection was shown by accumulation of viral IE proteins, particularly by high-level expression of IE2 and its SUMO-modified form during the late phases of infection (48 to 144 h) (Figure 3A). The efficacy of UV irradiation to inactivate viral gene expression was also confirmed by the absence of IE protein accumulation in UV-HCMV-infected cells (Figure 3A).To assess whether the temporal regulation of A20 protein level during HCMV infection at high MOIs reflects transcriptional change, cells were infected with HCMV or UV-HCMV at an MOI of 5, and the A20 mRNA level was measured at different time points by qRT-PCR analysis. The results showed that the A20 mRNA level was initially increased at the early phase of infection, but subsequently decreased at the later phase, and that UV-HCMV more profoundly activated the A20 transcription than intact virus (Figure 3B). These results correlate well with the changes in A20 protein levels, indicating that A20 expression during HCMV infection is primarily regulated at the transcriptional level. Furthermore, a comparison of the effects of HCMV and UV-HCMV infection suggests that, although A20 induction and termination occur during UV-HCMV infection, the newly synthesized viral gene product(s) may be involved in limiting A20 induction at high MOIs during HCMV infection.

### Evidence for epigenetic regulation of the A20 promoter during HCMV infection

To further investigate whether A20 transcription is epigenetically regulated, we examined changes in histone modification in the A20 promoter region after infection with HCMV and UV-HCMV. The results of ChIP assays performed at 24 and 48 h showed that the level of histone H4 acetylation, a marker for euchromatin, was slightly reduced by HCMV compared to mock-infection, whereas histone H4 acetylation was increased by UV-HCMV (Figure 3C). In addition, we measured the level of histone H3 tri-methylation, a marker for heterochromatin, and both HCMV and UV-HCMV showed increased levels as the infection progressed, but HCMV showed a more profound increase than UV-HCMV at 48 h (Figure 3D). These ChIP assay results indicate that HCMV infection led to more heterochromatin-favored histone modifications than did UV-HCMV at this high MOI, implicating a role for the viral gene product(s) in these changes. Taken together, the results of the qRT-PCR assays and the ChIP assays in virus-infected cells suggest that the expression of A20 is epigenetically controlled to limit its upregulation by the viral gene product(s) during HCMV infection.

### Role of A20 regulation in HCMV growth

**Figure 4:**
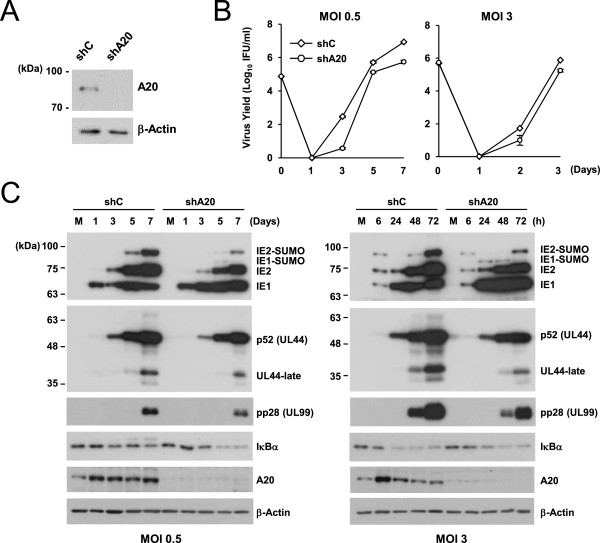
**Effects of knockdown on HCMV growth. (A)** HF cells expressing control shRNA (shC) and shRNA specific for A20 (shA20) were produced by lentiviral vectors. The levels of A20 and β-actin (as a loading control) were shown by immunoblotting. **(B and C)** Control (shC) and A20-knockdown (shA20) HF cells in 12-well plates were infected with HCMV at an MOI of 0.5 or 3. The growth curves shown represent the total numbers of infectious center-forming units (IFU) (averages of triplicate) produced in cell culture supernatants at the indicated sampling times **(B)**. Total cell lysates were prepared at indicated sampling times and the levels of viral immediate-early (IE1 and IE2), early (p52), and late (pp28) proteins and cellular IκBα, A20, and β-actin proteins were shown by immunoblotting **(C)**.

To access the significance of the fine regulation of A20 expression in HCMV growth, we investigated the effect of A20 depletion on HCMV growth. HF cells expressing control shRNA or shRNA specific for A20 were produced by lentiviral transduction. The result of immunoblotting showed a substantial reduction of A20 level in knockdown cells (Figure 4A). We compared the viral growth curves at low and high MOIs in control and A20-knockdown cells. The results showed that the virus titers in A20-knockdown cells were 15-fold less at 7 days at an MOI of 0.5 and 4-fold less at 3 days at an MOI of 3, compared to those in control cells (Figure 4B).We then examined the progression of infection by measuring the levels of viral proteins. We found at both MOIs that, although the level of IE1 was slightly higher in A20-knowdown cells than in control cells, the levels of IE2, p52 (an early protein encoded by UL44), and pp28 (a late protein encoded by UL99), were lower in A20-knockdown cells (Figure 4C). We also assessed NF-κB activity by determining the levels of IκBα. The results showed that at an MOI of 0.5, the IκBα levels were less efficiently reduced in control cells than in A20-knockdown cells, indicating that A20 induction after virus infection results in the lower NF-κB activity (Figure 4C, left panels). However, at an MOI of 3, the degradation of IκBα after virus infection was comparable between control and A20-knockdown cells, suggesting that under this high MOI condition, the NF-kB activity may be less effectively affected by A20 (Figure 4C, right panels). Taken together, these results suggest that the expression of A20 and its fine regulation during HCMV infection may be important for the efficient viral growth by affecting the accumulation of IE2 and subsequent expression of viral early and late proteins.

## Discussion

In this study, we evaluated the impact of HCMV infection on A20 expression, which plays a key role in regulating NF-κB signaling. We found that the A20 expression is increased upon HCMV infection. This is consistent with a previous report showing that the binding of HCMV glycoproteins to cellular receptors induces A20 transcription in human monocytes [4]. We also found that IE1 activates the A20 promoter. The direct activation of A20 promoter by IE1 is not surprising, since IE1 induces NF-κB activity and the A20 promoter contains two NF-κB binding sites. Thus, IE1 can exert opposite effects on NF-κB activity; it can increase NF-κB activity by directly activating the NF-κB subunit production [3,5-7] and also reduces NF-κB activity by indirectly inducing A20 expression. We found that A20 was moderately increased by IE1 expression alone and localized largely in the cytoplasm, whereas it was increased to a higher level by HCMV infection and accumulated at the cytoplasm and to a lesser extent the nucleus. Therefore, it is likely that other viral genes are also implicated in A20 upregulation and intracellular localization. Similar opposite effects on NF-κB activity have been observed during Kaposi’s sarcoma-associated herpesvirus (KSHV) infection [18]. The KSHV vFLIP protein promotes NF-κB activation by directly binding to NEMO and deregulates it by indirectly inducing A20 expression. A20 induction by vFLIP is thought to inhibit vFLIP-induced NF-κB activation. Therefore, both HCMV and KSHV appear to have a bi-directional regulatory mechanism to control NF-κB activity.

Notably, we found that the A20 regulation during HCMV is biphasic. The initial A20 upregulation was observed at the early phases of infection with both low and high MOIs. This biphasic regulation of A20 was more apparent with high MOIs, where the A20 expression was initially upregulated at the early phases of infection but downregulated during the late phases and these differences were controlled at the level of transcription. Importantly, the upregulation of A20 was much higher and sustained in UV-HCMV infection than in HCMV infection. Therefore, it appears that the viral gene products may be involved in limiting A20 upregulation. Intriguingly, we found that the effects of the newly synthesized viral gene product(s) on A20 regulation were different with different viral loads; viral gene expression promoted A20 upregulation with a low MOI, whereas it contributed to A20 downregulation with a high MOI. Further studies are necessary to investigate how A20 expression is differently regulated by different viral loads on a single cell level.

The exact role of A20 regulation during HCMV infection is not clear. Given the critical role of A20 in terminating canonical NF-κB signaling, HCMV may regulate A20 expression for elaborate modulation of the NF-κB activity during infection in a timely manner. The initial increase of A20 level after HCMV infection may just reflect the increase of NF-κB activity upon virus infection and this negative feedback regulation is to control excessive NF-κB activity, which may be harmful by provoking a robust immune response *in vivo*. This regulation appears to be necessary for the efficient progress of viral infection, since A20 depletion reduced viral growth by affecting the accumulation of IE2 and subsequent viral early and late proteins. Notably, the effect of A20 depletion on HCMV growth was more profound at a lower MOI, suggesting that reducing the NF-κB activity by A20 induction may be more critical for viral replication under low MOI conditions. Besides acting as a terminator in canonical NF-κB signaling, A20 also activates the non-canonical NF-κB pathway through NIK stabilization [17]. Therefore, it is complicated to expect the output NF-κB activity after A20 induction during HCMV infection. Further studies on the exact role of A20 during HCMV infection are warranted.

## Conclusion

In this study, we show that the A20 expression is biphasically regulated during HCMV infection. The A20 level was increased during the early phases of HCMV infection, but this induction was reduced at the late phases. Although A20 has been shown to be induced during HCMV entry, we also found that IE1 activates the A20 promoter through the upstream NF-κB sites. We also suggest that the downregulation of A20 induction at the late stages of infection may involve epigenetic controls of A20 transcription and that the newly produced viral gene product(s) may facilitate this process. Furthermore, we show that the A20 expression is required for efficient HCMV growth, highlighting the importance of the fine regulation of A20 gene expression in the virus replication cycle. This A20 regulation may affect NF-κB activity during HCMV infection or be critical in regulating unknown A20 targets or signaling that may be involved in HCMV growth.

## Materials and methods

### Cells and viruses

Human foreskin fibroblast **(**HF) and 293 T cells were grown in Dulbecco’s modified Eagle’s medium (DMEM) supplemented with 10% fetal bovine serum in a 5% CO2-humidified incubator at 37°C. The cell growth medium also contained 100 units/ml of penicillin and 100 μg/ml of streptomycin. The stock for the Towne virus and the production of UV-inactivated virus (UV-HCMV) were previously described [19]. Infectious center assays for the measurement of viral titers were also previously described [19].

### Plasmids

pSG5-based expression plasmids for HA-IE1 and HA-IE2 were described previously [20,21]. The A20 cDNA was cloned into a pENTR vector (Invitrogen). The retroviral vectors expressing A20 or IE1 were produced by transferring the A20 or IE1 cDNA from ENTR vectors to the pMIN-based destination vector using the LR Clonase (Invitrogen). Dr. Kun-Sang Chang (The University of Texas M. D. Anderson Cancer Center, Houston, USA) provided an A20(-95/+9)-luciferase reporter construct containing the A20 minimum promoter with two NF-κB sites [22,23]. In addition, Dr. Rivka Dikstein (The Weizmann Institute of Science, Rehovot, Israel) provided a set of pA20(-229/+12)-luciferase reporter plasmids containing wild-type or mutant NF-κB (mNF-κBs) sites. In these plasmids, the wild-type A20 promoter contained six Sp1 sites and two NF-κB sites, whereas the mNF-κBs A20 promoter contained mutant NF-κB sites [24]. pNFκB-Luc, a luciferase reporter plasmid that contains a synthetic promoter (herpes simplex virus thymidine kinase TATA-like promoter) with four copies of NF-kB sites from the MHC class II gene, was purchased from Clontech.

### Lentiviral and retroviral vectors

Lentiviral vector pLKO.1-TRC control expressing a non-hairpin control RNA (shC) was purchased from Addgene. Lentiviral vectors (on a pLKO.1 background) expressing shRNAs against A20 (shA20) (TRCN50959, 50961, and 218517) were purchased from Open Biosystems. Lentiviral stocks were prepared by cotransfecting 293 T cells with the lentiviral vectors together with packaging plasmids pCMV-DR8.91 expressing the gag-pol, tat, and rev proteins of human immunodeficiency virus (HIV) and pMD-G expressing the envelope G protein of vesicular stomatitis virus (VSV) [25]. For retroviral stocks, 293 T cells were cotransfected with the retroviral vectors (MIN, or MIN-IE1) together with packaging plasmids pHIT60 (Gag-Pol) and pMD-G (VSV-G) [26] using Omicsfect reagents (Omicsbio). Viral supernatants were harvested 48 h after transfection. HF cells were transduced by viral vectors in the presence of polybrene (7.5 μg per ml), and then were selected with puromycin (1 μg per ml) for lentiviral evctors or with G418 (0.5 mg/ml; Calbiochem) for retroviral vectors. For efficient knockdown A20, three lentiviral vectors expressing three different shRNAs were used together to transduce HF cells.

### Electroporation

HF cells were transfected via electroporation using the Neon Transfection System (Invitrogen). For each reaction, 3 × 10^5^ cells were suspended in 100 μl of resuspension (R) buffer and mixed with expression plasmids (up to 4 μg) in a 1.5-ml tube. After electroporation at 1,300 V and 40 ms, the cells were plated in six-well plates or chamber slides. 293 T cells were transfected using Omicsfect reagents (Omicsbio) according to the manufacturer’s instructions.

### Immunoblot analysis and antibodies

The standard procedures for immunoblot analysis were described previously [21]. The mouse monoclonal antibody (MAb) for A20 was purchased from Imgenex. Mouse MAbs for IE1/IE2, p52 (UL44), and pp28 (UL99), rat anti-HA MAb (3 F10) conjugated to peroxidase, and mouse MAb against β-actin were described previously [21]. Rabbit polyclonal Ab (PAb) raised against purified His-IE1 were described previously [20,21]. Mouse MAb for IκBα was purchased from AbFrontier. Secondary Abs for immunoblot analysis were obtained from Jacson ImmunoResearch Laboratories, Inc.

### Indirect immunofluorescence assay (IFA)

Cells were fixed for 5 min in 1% paraformaldehyde and permeabilized in cold 0.2% Triton X-100, or fixed for 5 min in cold and rehydrated in cold PBS and then incubated with appropriate Abs in PBS for 1 h at 37°C and then incubated with fluorescein isothiocyanate (FITC)-labeled or rhodamine/red X-coupled donkey immunoglobulin G (IgG) (Jackson ImmunoResearch Laboratories, Inc.). For double-labeling, two different Abs were incubated together. The slides were examined and photographed with a Carl Zeiss Axiophot microscope.

### RNA isolation and quantitative real-time reverse-transcription PCR (RT-PCR)

Total RNA was isolated from 2 × 10^5^ cells using TRIzol reagent (Invitrogen) and a MaXtract High Density Tube (Qiagen). cDNAs were synthesized using the random hexamer primers in the SuperScript III system (Invitrogen). Quantitative real-time PCR was performed using the SYBR green PCR core reagents (Applied Biosystems) and ABI Prism SDS software. The primers used to amplify A20 were 5’-CACACAAGGCACTTGGATCC-3’ (forward) and 5’-TCCCCAGGAGTCCGTGCAGC-3’ (reverse).

### Chromatin immunoprecipitation (ChIP) assays

ChIP assays were carried out using a kit (Upstate Biotechnology, Inc.). HF cells (6 × 10^6^) mock-infected or infected with HCMV were fixed with 1% formaldehyde for 10 min at 24 h after infection and then lysed with the lysis buffer provided in the kit. ChIP assays were performed with 5 μg of anti-acetyl-histone H4 Ab (Millipore), anti-trimethyl-histone H3 (K9) Ab (Millipore), or control IgG. One-sixth of the lysates were reserved for quantitation of the DNA present in different samples prior to immunoprecipitation. Relative changes in precipitated DNA were calculated via PCR and agarose gel electrophoresis. The primer sequences used for amplification of the A20 promoter region were 5’-CAGCCCGACCCAGAGAGTCAC-3’ (forward) and 5’-CTCCGGGCCCCGCGATCC-3’ (reverse). The PCR program used involved 30 amplification cycles described as follows: 95°C for 30 s, 62°C for 30 s, and 72°C for 45 s.

### Luciferase reporter assay

Cells were lysed using three freeze-thaw steps in 100 μl of 0.25 M Tris–HCl (pH 7.9) containing 1 mM dithiothreitol. Subsequent procedures were performed as previously described [21]. A TD-20/20 luminometer (Turner Designs) was used for the 10-s assay of the photons produced.

## Competing interests

The authors declare that they have no competing interests.

## Authors’ contributions

SYG, YEK, THH, and JHA designed the experiments. SYG, YEK, and KMK performed the experiments. SYG, YEK, and JHA drafted the manuscript. All authors read and approved the final manuscript.
